# Clinical Performance of Immediately Placed and Restored Implants With a Novel Design in the Esthetic Zone. A 3‐Year Follow‐Up of Prospective Case Series

**DOI:** 10.1111/clr.14438

**Published:** 2025-04-06

**Authors:** Frank Schwarz, Georgina Trimpou, Alexa Montada, Karina Obreja, Puria Parvini, Amira Begić

**Affiliations:** ^1^ Department of Oral Surgery and Implantology Goethe University, ZZMK Carolinum Frankfurt Germany

**Keywords:** clinical study, immediate implant placement, immediate restoration

## Abstract

**Aim:**

To assess the medium‐term implant success and survival rates of immediately placed and restored implants with a novel design in the esthetic zone.

**Materials and Methods:**

A total of *n* = 20 patients had received immediately placed tapered, two‐part implants with a progressive thread design (PL) (*n* = 20) and an immediate “non full‐functional” loading (patient‐specific abutments) for single tooth replacements in the anterior maxilla. Implant survival and success (e.g., bleeding on probing—BOP, probing pocket depth—PD, mucosal recession—MR) were assessed at 24 and 36 months following final restoration (i.e., 12 weeks after implant placement). Patient‐reported outcomes were evaluated at 36 months.

**Results:**

A total of four patients were lost to follow‐up, resulting in 16 patients included in the analysis. At 24 and 36 months, implant survival amounted to 100%, respectively. Non‐significant changes to baseline were noted at 24 and 36 months for mean BOP (9.37 (29.7)%, 9.38 (26.5)%), PD (−0.45 (0.88)mm, −0.34 (0.74)mm), and MR (0.0 (0.0)mm, 0.0 (0.02)mm) values. Pink esthetic score—PES values amounted to 13.0 (1.22) and 12.62 (1.42) at 24 and 36 months. Technical and mechanical complications were not observed. Patients expressed an overall high satisfaction at 36 months.

**Conclusions:**

The presented immediacy protocol was associated with high survival and success rates as well as a high patient satisfaction in the medium‐term.

## 1 Introduction

Immediate implant placement (IIP) has become an integral therapeutic component for single‐tooth replacements in the esthetic zone (Cosyn and Blanco 2023). Two systematic reviews and meta‐analyses have pointed to similar survival rates when IIP was compared with conventional delayed implant placement. Likewise, both approaches were associated with similar clinical (i.e., probing depths) and esthetic outcomes (Cosyn et al. 2019; Garcia‐Sanchez et al. 2022). Nevertheless, clinical cases for IIP should be carefully selected. Among a variety of potential factors, the absence of acute infections, a sufficient bone dimension to ensure primary implant stability, the presence of a favorable morphology of the extraction socket, and a proper three‐dimensional implant positioning appear to be some of the most crucial prerequisites to obtain the most predictable treatment outcomes (Cosyn and Blanco 2023; Tonetti et al. 2019). Likewise, it has been demonstrated that the treatment outcomes following IIP were also influenced by the surgical procedure. While it is unclear whether there is a clinical difference between a flap and flapless approach (Pitman et al. 2023), grafting the gap between the implant and socket walls, as well as soft tissue volume grafting, have been shown to positively influence horizontal buccal bone resorption and mid‐facial soft tissue levels (Seyssens et al. 2021, 2022).

Apart from IIP, contemporary immediacy concepts also encompass a non‐functional immediate provisionalization (IP), namely type 1/A implant placement and loading/restoration protocol (Morton et al. 2023). This may offer the advantage of overcoming a removable provisional prosthesis (Gallucci et al. 2014) and facilitate early bone healing by a controlled mechanical stimulus (Suzue et al. 2022). IIP and IP for single implants in the esthetic zone can be considered a well‐established treatment solution with high survival rates and predictable esthetic outcomes, which allows for reducing the overall treatment time and patients' morbidity (Obreja et al. 2022; Trimpou et al. 2022; Wittneben et al. 2023).

Likewise, another systematic review and meta‐analysis indicated that IP had apparently no negative influence on implant survival and success but contributed to a stabilization of the mid‐facial mucosal margin (Pitman et al. 2022).

In the same context, recently presented data from our group applying both IIP and IP, using a progressive‐type two‐piece implant considering the “one abutment‐one time principle” in the aesthetic zone, showed high survival and success rates at 12 months (Trimpou et al. 2022).

The present follow‐up of a prospective observational study aimed at evaluating implant survival and success of this immediacy concept over a period of 3 years.

## 2 Materials and Methods

### 2.1 Study Design and Participants

This prospective observational study reports on a total of 16 patients having received an immediate type‐1 implant placement and non‐full functional loading/immediate temporary (type‐1/A) on a final patient‐specific abutment (one abutment‐one time concept) for a single tooth replacement in the anterior maxilla using a two‐piece implant with a novel, progressive implant thread design (Tables 1 and 2). The short‐term outcomes of *n* = 20 patients (*n* = 20 implants) that were treated in the Department of Oral Surgery and Implantology at the Goethe University Frankfurt, Germany between March 2019 and May 2021 have been presented previously (Trimpou et al. 2022).

**TABLE 1:** Patient characteristics.

Patient age mean	47.6 (14.2) years; range: 21–67 years
Female/male	*n* = 10/6
ASA physical status classes	I (normal healthy) = 11 II (mild systemic disease) = 5
Smoking	None = 10 < 10 cigarettes per day = 6

**TABLE 2:** Reasons for tooth extraction and implant site characteristics.

Reason for tooth extraction	Caries = 2 Endodontic lesion = 6 Fracture = 11 Persistent deciduous tooth = 1
Defect dimension	Width = 0.62 (1.70) mm Length = 1.43 (3.96) mm
Mucosal thickness	1.37 ± 0.72 mm
Distance implant—adjacent tooth	Mean distal/mesial = 2.54 (0.80) mm
Implant diameters	3.3 mm = 3 3.8 mm = 4 4.3 mm = 7 5.0 mm = 2
Implant length	11 mm = 2 13 mm = 10 16 mm = 4
Insertion torque	36.12 (7.54) Ncm

**FIGURE 1:**
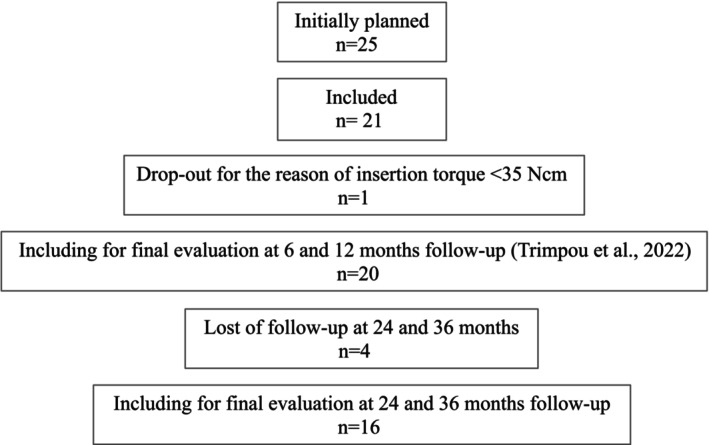
Flowchart depicting patient recruitment.

Due to disruptions caused by the COVID‐19 pandemic, a total of *n* = 4 patients were lost to regular follow‐up visits at 24 (*n* = 0) and 36 (*n* = 4) months, thus resulting in a total of *n* = 16 patients being analyzed for the present study (Figure 1). The study was in accordance with the Helsinki Declaration of 1964 (as revised in 2000) and the protocol including the extended follow‐up period was approved by the ethics committee of the Goethe University, Frankfurt, Germany, and prospectively registered via the Internet Portal of the German Clinical Trials Register (DRKS00016500). Before participating, every patient received a comprehensive explanation of the study procedures and provided signed consent. The present reporting considered the checklist items as proposed in the STROBE statement (von Elm et al. 2014).

### 2.2 Inclusion Criteria

The patient inclusion and exclusion criteria have been presented in detail previously (Trimpou et al. 2022).

In brief, the inclusion criteria were the following: (1) males and females; age of 18–65 years, (2) needing a single tooth replacement in the maxilla (14–24 FDI), (3) absence of acute or chronic infections at the adjacent teeth, (4) Single tooth gaps with adjacent natural teeth, (5) natural opposing dentition or restored with fixed restoration, (6) stable occlusal relationship.

To be included in the present analysis, each participant had to complete all follow‐up visits and recordings of all primary and secondary outcome measures at 24 and 36 months following the initial surgery.

### 2.3 Clinical Assessment of Primary and Secondary Outcomes

The primary outcome considered implant success as evaluated by the peri‐implant tissue health status (Tonetti et al. 2023), including the clinical assessment of bleeding on probing (BOP), probing pocket depth (PD), and mucosal recession (MR).

The secondary outcomes included implant survival (i.e., presence of the implant in situ at the 24‐ and 36 months follow‐up examinations), plaque index, pink esthetic score—PES (scoring from 2 to 0) (Furhauser et al. 2005), technical and mechanical complications as well as patient‐reported outcome measures (PROM's) (A.B., G.T.) (Trimpou et al. 2022).

Clinical measurements were recorded as follows: (1) plaque index (PI) (Loe 1967), (2) BOP, evaluated as present if bleeding was evident within 30 s after probing or absent if no bleeding was noticed within 30 s after probing, (3) PD measured from the mucosal margin to the bottom of the probeable pocket, (4) MR measured from the crown margin to the mucosal margin. All measurements were recorded at six aspects per implant: mesiovestibular (mb), midvestibular (b), distovestibular (db), mesiooral (mo), midoral (o), and distooral (do). All measurements were performed by two calibrated and experienced investigators (A.B., K.O.).

The presence of peri‐implant diseases (i.e., 24 and 36 months) was evaluated based on established case definitions (Herrera et al. 2023; Schwarz et al. 2018): peri‐implant mucositis—presence of BOP and/or suppuration with or without increased PD, and peri‐implantitis—presence of BOP and/or suppuration with increased PD and presence of radiographic bone loss (i.e., baseline to 6 and 12 months). Peri‐implant health was defined as an absence of BOP and/or suppuration on gentle probing, no increase of PDs, and an absence of radiographic bone loss (i.e., baseline to 6 and 12 months) (Berglundh et al. 2018). Implant success was defined by the absence of peri‐implantitis.

As described in the previous paper, radiographs were just taken if clinically indicated (i.e., in the presence of clinical signs suggesting the presence of peri‐implantitis or mechanical/technical complications), and not as a routine procedure (Trimpou et al. 2022).

Mechanical complications considered all the events affecting the integrity of the implant or of the abutment. Technical complications considered all the events affecting the integrity of the implant‐supported restoration (i.e., crown).

PROM's were assessed at 36 months by a questionnaire on (1) prosthesis comfort, (2) prosthesis appearance, (3) chewing, (4) tasting ability, (5) prosthesis fit, and (6) overall satisfaction, with scores ranging from 1 (very satisfactory) to 5 (unsatisfactory) (Trimpou et al. 2022) (Supporting Information—S1). Prior to the clinical investigation at the 36 months follow‐up, patients completed the aforementioned questionnaires at the clinic in the absence of the treating practitioner.

### 2.4 Sample Size Calculation

A sample size calculation was not feasible due to insufficient reference data on this immediate protocol using a specific one abutment–one time concept in the existing literature.

### 2.5 Surgical and Prosthetic Procedures

The surgical and prosthetic procedures have been presented in detail previously (Trimpou et al. 2022).

In brief, a flapless atraumatic extraction of the respective teeth was accomplished under local anesthesia. Following a meticulous granulation tissue removal and inspection of the integrity of the socket walls using a periodontal probe, platform‐switched tapered, two‐part implants with a progressive thread design (CAMLOG PROGRESSIVE‐LINE, CAMLOG Biotechnologies GmbH) (PL) were placed in a subcrestal position (2–3 mm) and at a distance of 1.5–2.0 mm to the vestibular comfort zone (Ramanauskaite and Sader 2022). The aforementioned progressive‐type two‐piece implant (PL) combines different thread designs that extend from the upper parallel section to the apex of the lower tapered section, providing adequate primary implant stability (Krischik et al. 2021). The upper, crestal section of the self‐tapping two‐part implant is characterized by a grooved thread design and a 0.4 mm high machined implant neck.

The primary stability was assessed by insertion torque measurements (Implantmed, W&H, Bad Reichenhall, Germany). A natural bone mineral was used for peri‐implant gap grafting (Bio‐Oss spongiosa, Geistlich Pharma AG).

Subsequently, intraoral scans were taken (Cerec Omnicam; Dentsply Sirona) to assess the implant position (CAMLOG ScanPosts for Sirona Scanbody CAMLOG Biotechnologies GmbH) and the opposite jaws.

**FIGURE 2:**
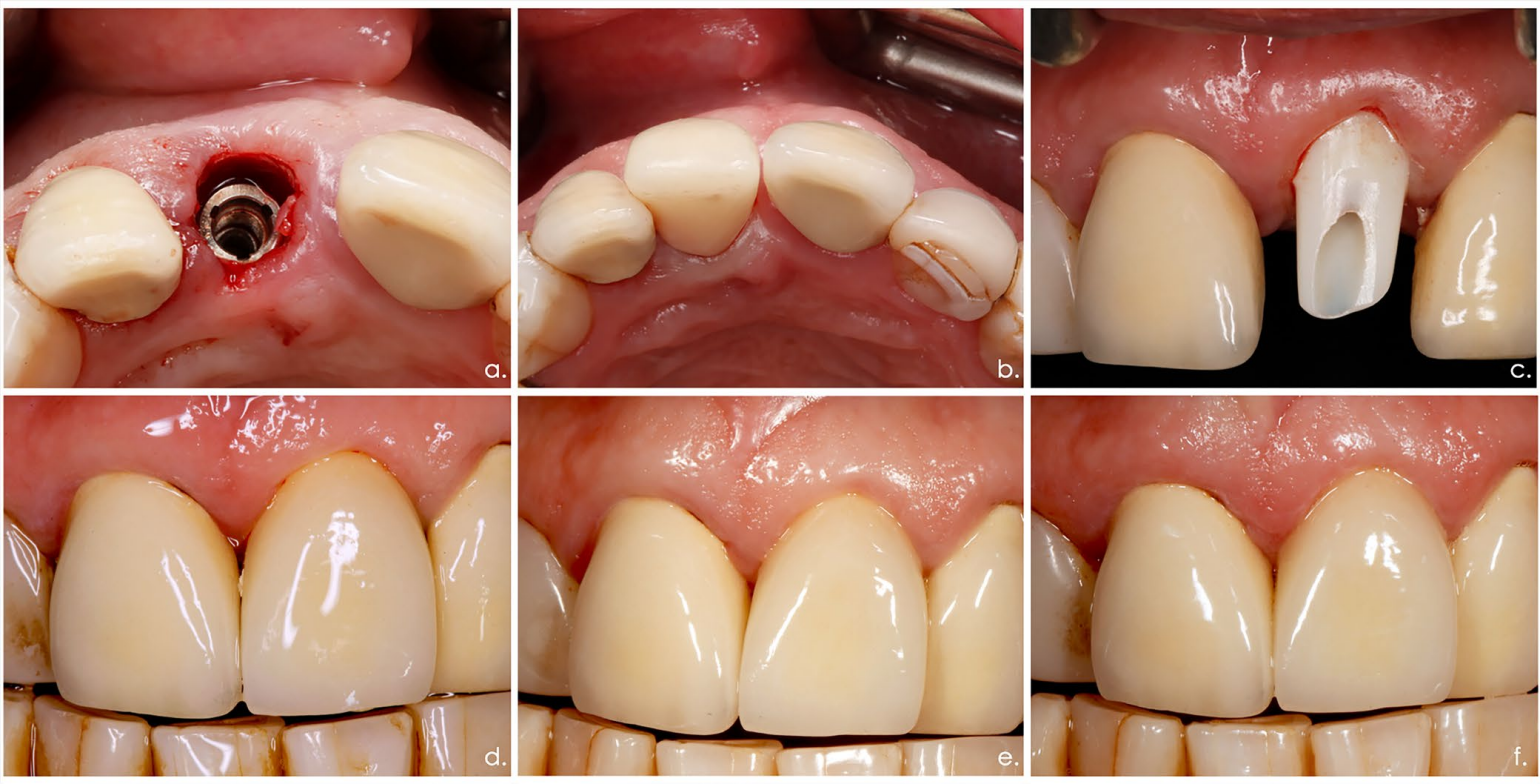
Surgical and prosthetic steps of the treatment protocol. (a) Immediate implant placement following tooth extraction region 021. (b) Immediate connection of the final abutment and provisional crown in non‐functional occlusion. (c) Clinical situation of the definitive hybrid abutment at 3 months. (d) Clinical situation following cementation of the final crown (Baseline). (e) Clinical situation at 24 months. (f) Clinical situation at 36 months.

Four prosthetic components were fabricated within 2 h, including (1) a definitive patient‐specific hybrid abutment (i.e., CAMLOG Ti Base CAD/CAM PS CAMLOG Biotechnology GmbH + bonded (Multilink Hybrid Abutment, Ivoclar‐Vivadent, Ellwangen, Germany) mesostructure (IPS e.max CAD Cerec/In Lab, Ivoclar‐Vivadent)), (2) a milled (Katana Zirconia Block; Kuraray, Hattersheim, Germany) and rapidly sintered (CEREC SpeedFire Dental furnace, Dentsply Sirona) framework for the definitive crown, (3) a milled provisional crown (Vita CAD‐Temp monoColor, Vita, Bad Säckingen, Germany) without functional occlusion, and (4) a conventionally manufactured abutment replica for the extraoral cementation technique. The definitive hybrid abutment was mounted with the recommended insertion torque. The provisional crown was cemented using the extraoral cementation technique with a temporary cement (Temp Bond; Kerr GmbH, Herzogenrath, Germany). The occlusion was visually checked with the use of a articulation paper. Patients were instructed to adhere to a soft diet for a period of 12 weeks. After 3 months, the final full‐ceramic crowns (Cercon Cream Kiss, Degudent) were cemented extraorally (Temp Bond) using an abutment replica in full occlusion. (Figure 2a–d).

### 2.6 Postoperative Care

Postoperative care involved professional supramucosal−/gingival implant and tooth cleaning as well as an oral hygiene reinforcement at 24 and 36 months. Additional hygiene sessions were provided according to individual needs.

### 2.7 Statistical Analysis

The statistical analysis of the pseudonymised data sets was accomplished using a commercially available software program (IBM SPSS Statistics 29.0, IBM Corp., Armonk, NY, USA).

Mean values, standard deviations, medians, 95% confidence intervals (CI) and frequency distributions were calculated for all clinical parameters. For the changes in clinical parameters (i.e., baseline—24 months and 36 months), interquartile ranges (IQR) were also calculated. Data were checked for normality using the Shapiro‐Wilk‐test. Within group comparisons of mean BOP, PD, PI, and PES values from baseline to 24 and 36 months were accomplished using the Friedman test.

## 3 Results

### 3.1 Implant Survival

At 24 and 36 months, the survival rate amounted to 100%, as indicated by an absence of clinical mobility at all implant sites investigated.

### 3.2 Clinical Measurements

Mean and median BOP, PD, MR, PI, and PES values measured at baseline, 24, and 36 months are summarized in Tables 3, 4, 5. The respective changes in clinical parameters between baseline and 24 and 36 months are presented in Tables 6 and 7.

**TABLE 3:** Clinical parameters measured at baseline (i.e., final restoration) (*n* = 16 patients).

	BOP	PD	MR	PI	PES
Mean	6.25	2.93	0.01	0.00	12.81
SD	10.31	0.87	0.06	0.00	1.16
Median	0.00	2.91	0.00	0.00	13.00
95% CI	0.7;11.75	2.46;3.40	−0.01;0.04	0.00;0.00	12.19;13.43

**TABLE 4:** Clinical parameters measured at 24 months (*n* = 16 patients).

	BOP	PD	MR	PI	PES
Mean	15.63	2.47	0.01	0.13	13.0
SD	25.43	0.47	0.06	0.25	1.22
Median	0.00	2.41	0.00	0.00	13.50
95% CI	2.0;29.18	2.22;2.73	−0.01;0.04	−0.003;0.27	12.34;13.65

**TABLE 5:** Clinical parameters measured at 36 months (*n* = 16 patients).

	BOP	PD	MR	PI	PES
Mean	15.63	2.59	0.02	0.02	12.62
SD	22.33	0.43	0.08	0.08	1.42
Median	0.00	2.66	0.00	0.00	13.00
95% CI	3.72;27.53	2.36;2.82	−0.02;0.06	−0.02;0.06	11.86;13.38

**TABLE 6:** Changes (d) in clinical parameters between baseline and 24 months (*n* = 16 patients).

	dBOP	dPD	dMR	dPI	dPES
Mean (SD)	9.37 (29.7)	−0.45 (0.88)	0.0 (0.0)	0.13 (0.25)	0.18 (1.19)
Median	0.0	−0.66	0.0	0.00	0.5
IQR	0.46	0.96	0.00	0.17	1.75
95% CI	−6.50; 25.25	−0.92; 0.01	0.0; 0.0	−0.003; 0.27	−0.44; 0.82
*p* *	0.86	0.01	0.79	0.18	0.37

*Note:* Within group comparison (i.e., baseline—24 months).

* Friedman test.

**TABLE 7:** Changes (d) in clinical parameters between baseline and 36 months (*n* = 16 patients).

	dBOP	dPD	dMR	dPI	dPES
Mean	9.38 (26.5)	−0.34 (0.74)	0.0 (0.02)	0.02 (0.08)	−0.18 (1.46)
Median	0.0	−0.33	0.0	0.00	−0.25
IQR	0.42	0.75	0.00	0.00	1.75
95% CI	−4.75; 23.50	−0.74; 0.05	−0.005; 0.01	−0.02; 0.06	−0.95; 0.57
*p* *	0.53	0.13	0.79	0.79	0.72

*Note:* Within group comparison (i.e., baseline—36 months).

* Friedman test.

Over the entire follow‐up observation period of 3 years, all patients revealed a good level of oral hygiene, as evidenced by median PI scores of 0.00 at respective implant sites (Tables 3, 4, 5). At 24 and 36 months, mean changes in BOP amounted to 9.37 (29.7)% and 9.38 (26.5)%, respectively, and failed to reach statistical significance (*p* = 0.86, *p* = 0.53, Friedman test) (Tables 3, 4, 5, 6, 7). Mean PD scores slightly decreased by 0.45 (0.88) mm and 0.34 (0.74) mm at 24 and 36 months, respectively (*p* = 0.01, *p* = 0.13, Friedman test) (Tables 3, 4, 5, 6, 7). Changes in mean MR values were minimal and did not reach statistical significance (*p* = 0.79, *p* = 0.79, Friedman test) (Tables 3, 4, 5, 6, 7). The mean PES values amounted to 13.0 (1.22) and 12.62 (1.42) at 24‐ and 36‐months, respectively. The respective changes to baseline amounted to 0.18 (1.19) and −0.18 (1.46), respectively (*p* = 0.37, *p* = 0.72, Friedman test) (Figure 2e,f; Tables 3, 4, 5, 6, 7).

### 3.3 Incidence of Peri‐Implant Diseases

The frequency distribution of peri‐implant diseases at 24 and 36 months is summarized in Table 8. According to the given case definitions, the incidence of peri‐implant mucositis and peri‐implantitis amounted to 31.30% and 0.0% at 24 months and to 25.00% and 0.0%, respectively.

**TABLE 8:** Incidence of peri‐implant diseases at 24 and 36 months.

	Diagnosis			Total
	0	1	2	
24 months				
Count	11	5	0	16
%	68.8%	31.2%	0.0%	100.0%
36 months				
Count	12	4	0	16
%	75.0%	25.0%	0.0%	100.0%

*Note:* Diagnosis: 0 = peri‐implant health; 1 = peri‐implant mucositis; 2 = peri‐implantitis.

### 3.4 Incidence of Technical and Mechanical Complication

Abutment loosening was noted in one patient at 24 months. Throughout the observation period of 36 months, no further technical or mechanical complications were noted.

### 3.5 PROM's

The mean values for the evaluated questionnaire items at 36 months amounted to 1.13 (SD 0.34; min. 1; max. 2) for prosthesis comfort, 1.06 (SD 0.25; min. 1; max. 2) for prosthesis appearance, 1.19 (SD 0.40; min. 1; max. 3) for chewing ability, 1.25 (SD 0.44; min. 1; max. 3) for tasting ability, 1.31 (SD 0.70; min. 1; max. 3) for prosthesis fit, and 1.13 (SD 0.34; min. 1; max. 2) for overall satisfaction.

## 4 Discussion

The present follow‐up of a prospective observational study aimed at evaluating implant survival and success of an immediacy concept for single‐tooth replacements in the esthetic zone over a period of 3 years. A basic component of this concept was the prosthetic protocol used for IP, which followed an established procedure (Parvini et al. 2020) and the “one abutment‐one time concept” (Tallarico et al. 2018). The latter was proven to overcome relevant hard and soft tissue changes that may occur as a consequence of repeated healing abutment exchanges (Becker et al. 2012; Tallarico et al. 2018).

Over the medium‐term follow‐up period of 36 months, the presented immediacy concepts were associated with high survival rates of 100% and non‐significant changes in mean BOP (9.38 (26.5)%), PD (−0.34 (0.74) mm), and MR (0.0 (0.02) mm) values when compared with the respective values noted at baseline (i.e., at final restoration). Furthermore, IIP and IP of PL implants were associated with high PES values amounting to 13.0 (1.22) and 12.62 (1.42) at 24 and 36 months, respectively. The absence of any technical or mechanical complications also contributed to the high level of satisfaction commonly reported by all patients.

The noted survival and success rates are basically within the range of previously published data on IIP and IP after a period of 24 to 48 months (Crespi et al. 2018; Gastaldi et al. 2017; Shibly et al. 2010).

In particular, one previous study (Crespi et al. 2018) reported on a 48‐month follow‐up of flapless IIP + IP in the anterior maxilla. A gap filling was performed at all implant sites (*n* = 30 patients/*n* = 30 implants) and the provisional restoration was reported to be in occlusion. The implant survival amounted to 100% with mean PD and BOP scores of 2.71 (0.16) mm and 42.0 (9.0) %, respectively. MR and PES scores have not been reported. However, vertical and horizontal midfacial soft tissue changes were minimal and amounted to −0.30 (0.58) mm and −0.32 (0.53) mm (Crespi et al. 2018).

Gastaldi et al. (2017) reported on a 24‐month follow‐up of flapless IIP + IP in the anterior maxilla and mandible. A gap filling was not provided and IP was under contact (*n* = 25 patients/*n* = 25 implants). The implant survival amounted to 100% with minimal marginal bone level changes of −0.10 (0.09) mm. PD, MR, BOP, and PES scores have not been reported (Gastaldi et al. 2017).

Likewise, (Shibly et al. 2010) reported on a 24‐month follow‐up of IIP + IP in the anterior and posterior maxilla and mandible. A gap filling was provided at all implant sites following flap elevation (*n* = 30 patients/*n* = 30 implants). One out of 30 implants was lost and marginal bone level changes amounted to 1.19 (0.26) mm. PD, MR, BOP, and PES scores as well as midfacial soft tissue changes have not been reported (Shibly et al. 2010).

Discrepancies noted between the aforementioned studies and the present analysis might be due to differences in the surgical‐ (e.g., integrity of the extraction socket, implant types, required insertion torques, gap filling) and/or prosthetic components (e.g., one‐abutment‐one time concept, provisional vs. functional loading) of the respective immediacy protocols (Crespi et al. 2018; Garcia‐Sanchez et al. 2021; Gastaldi et al. 2017; Shibly et al. 2010; Trimpou et al. 2022; Wittneben et al. 2023).

However, the aforementioned studies, with the exception of our previously published study (Trimpou et al. 2022), did not use the “one abutment‐one time” concept. Other studies on the “one abutment‐one time” in the esthetic zone only focused on the evaluation of marginal bone level and biological complications (Grandi et al. 2014) making the comparison more difficult.

The one‐abutment‐one time concept can benefit the stability of the midfacial soft tissue position and bone level (Molina et al. 2017; Yuan et al. 2023).

Moreover, the biological complications associated with cement remnants were not observed in our study, probably due to the use of a previously proven framework of extraoral cementing (Obreja et al. 2022).

In this context, it must also be emphasized that the presence of a compromised socket, particularly when there is > 50% loss of one or more walls, may result in increased PD‐ and decreased PES values (Tonetti et al. 2019). However, this observation could not be confirmed when evaluating the changes in BOP and PES values and, consequently, the incidence of peri‐implant diseases, mostly due to the inclusion of only minor to moderate buccal dehiscence‐type defects.

Furthermore, it must be noted that a potential drawback of the present analysis was the small sample size and the lack of any control group. In the aforementioned studies, IIP had been evaluated with or without IP (Crespi et al. 2018; Gastaldi et al. 2017; Shibly et al. 2010). While two of the studies reported on similar outcomes in both groups (Gastaldi et al. 2017; Shibly et al. 2010), one study noted more stable midbuccal mucosal margins and radiographic bone levels following IP (Crespi et al. 2018).

Connective tissue grafting (CTG) in combination with IIP + IP can favor the stabilization of the peri‐implant soft tissue in the esthetic zone (Atieh and Alsabeeha 2020). On the contrary, often no beneficial results are also constantly reported, but with a significant increase in morbidity due to a second surgical site (van Nimwegen et al. 2018). The maintenance of midfacial gingival margin and papilla height in a recent study further supports the “one abutment—one time concept” in IIP + IP without the necessity of additional CTG after 6 months (Yuan et al. 2023). Indeed, when evaluating the aesthetic outcomes with PES, additional CTG was not demonstrably superior to the non‐grafting group (Obreja et al. 2022; van Nimwegen et al. 2018).

The present study suffers from limitations, such as the lack of a sample size calculation, the lack of a control group as well as the heterogeneity of both intact and dehiscence‐affected sockets. Despite the aforementioned limitations, the study provides valuable results in terms of external validity regarding the indication of the one abutment‐one time concept for IIP and IP. However, further investigations based on this protocol, with sufficient statistical power, are required to properly support the current findings.

In conclusion and within the limitations of a single‐arm observational case series, the presented immediacy protocol was associated with high survival and success rates at 3 years.

## Author Contributions

**Frank Schwarz:** conceptualization, supervision, funding acquisition, writing – original draft, methodology, validation, visualization. **Georgina Trimpou:** conceptualization, methodology, writing – review and editing, formal analysis, project administration, data curation, funding acquisition, investigation. **Alexa Montada:** methodology, formal analysis, project administration, data curation, writing – original draft, writing – review and editing. **Karina Obreja:** methodology, formal analysis, writing – review and editing, data curation, investigation. **Puria Parvini:** data curation, formal analysis. **Amira Begić:** writing – original draft, writing – review and editing, methodology, data curation, formal analysis, investigation.

## Conflicts of Interest

Frank Schwarz and Georgina Trimpou have received a research grant for the present study from the Oral Reconstruction Foundation. Frank Schwarz has received lecture fees from the Oral Reconstruction Foundation. The authors declare no conflicts of interest related to this study.

## Supporting information


**Data S1.** Supporting Information.

## Data Availability

The data that support the findings of this study are available from the corresponding author upon reasonable request.
