# Supplementary material for: Clinical Performance of Immediately Placed and Restored Implants With a Novel Design in the Esthetic Zone. A 3‐Year Follow‐Up of Prospective Case Series
Source: Clin Oral Implants Res. 2025 Apr 6;36(7):903–11. doi: 10.1111/clr.14438 (PMC12230904; doi:10.1111/clr.14438)
Supplement: Supplementary file 1 — Data S1. Supporting Information. [file CLR-36-903-s001.pdf]

Patient- reported outcomes

Patient satisfication			Visit
	Subject No.	Visit Date	

Study visit

□ Final restoration □ 1-year follow-up □ 3-year follow-up □ 5-year follow-up

How satisfied are you with your implant restoration?

**P1**	**Comfort**	□ very satisfactory	□ satisfactory	□ middle	□ unsatisfactory	□ very unsatisfactory
**P2**	**Appearance**	□ very satisfactory	□ satisfactory	□ middle	□ unsatisfactory	□ very unsatisfactory
**P3**	**Chewing**	□ very satisfactory	□ satisfactory	□ middle	□ unsatisfactory	□ very unsatisfactory
**P4**	**Fit**	□ very satisfactory	□ satisfactory	□ middle	□ unsatisfactory	□ very unsatisfactory
**P5**	**Tasting ability**	□ very satisfactory	□ satisfactory	□ middle	□ unsatisfactory	□ very unsatisfactory
**P6**	**Overall satisfaction**	□ very satisfactory	□ satisfactory	□ middle	□ unsatisfactory	□ very unsatisfactory
